# It matters what we do: Relationships between forms of media use and life satisfaction of adolescents

**DOI:** 10.1093/eurpub/ckac131.467

**Published:** 2022-10-25

**Authors:** T Schloemer, I Rocco, P Kolip

**Affiliations:** 1 Department of Prevention and Health Promotion, Bielefeld University, Bielefeld, Germany; 2 Department of Applied Health Sciences, Hochschule für Gesundheit, Bochum, Germany; 3 Care and Public Health Research Institute, Maastricht University, Maastricht, Netherlands; 4 School of Statistical Sciences, Sapienza University of Rome, Rome, Italy

## Abstract

**Background:**

Electronic media communication is firmly anchored in the leisure time of adolescents. Adolescents use electronic devices (ED) as a means of interpersonal communication with friends, such as text messaging, for entertainment in the form of receptive communication, such as watching movies, in the form of interactive communication, such as playing computer games, and for other purposes, such as homework. We aimed to identify how these communication forms are related to and interact on life satisfaction (LS) of girls and boys.

**Methods:**

We conducted multivariable linear regression analysis with the dataset of 5,961 adolescents aged 11, 13 and 15 years, obtained from the German Health Behaviour in School-aged Children (HBSC) survey of 2013/2014. Separate statistical analyses were performed for girls and boys, including statistical interactions. We controlled for age, family affluence and family structure.

**Results:**

Interpersonal communication with friends has a positive relationship with LS in boys (β = 0.12, t = 3.19, p = 0.001), but no effect in girls. Whereas other communication forms have a negative main effect on life satisfaction in girls (entertainment β = -0.1, t = -3.79, p < 0.001; gaming β = -0.09, t = -4.12, p < 0.001; using the ED for other purposes β = -0.04, t = -2.26, p = 0.024), we found no association of entertainment with LS for boys. The negative effect of gaming in boys is conditional on the level of using the ED for other purposes: the results show a reinforcing statistical interaction (β = 0.02, t = 3.24, p = 0.001).

**Conclusions:**

The results demonstrate the relevance of considering the differences in associations between single forms of communication and LS for boys and girls separately. This confirms our theoretical focus on a communication-centred approach. Moreover, it is of high relevance to identify potentially enabling and harmful media communication and to understand adolescents’ perspectives on these forms of communication.

**Key messages:**

• When examining associations between media use and life satisfaction among adolescents, form of communication and gender should be taken into account.

• Investigating interactions of different forms of communication can help to better understand their influence on adolescents’ life satisfaction.

